# Method Development and Clinical Utility for Simultaneous Measurement of 21-Deoxycortisol, 17-Hydroxyprogesterone, Cortisol, and Cortisone Levels in Human Plasma Using UHPLC-MS/MS

**DOI:** 10.1155/adpp/3859670

**Published:** 2025-01-17

**Authors:** Syed N. Alvi, Saleh Al dgither, Ali Al-Odaib

**Affiliations:** ^1^Environmental Health Program, Cell Biology Department, King Faisal Specialist Hospital & Research Center, P.O. Box 3354, MBC-03, Riyadh 11211, Saudi Arabia; ^2^Research Administrative Operations, Research and Innovation, King Faisal Specialist Hospital & Research Center, P.O. Box 3354, MBC-03, Riyadh 11211, Saudi Arabia

**Keywords:** 21-deoxycortisol and 17-hydroxyprogesterone, cortisol, cortisone, plasma, UHPLC-MS/MS

## Abstract

A simple and efficient validated assay for quantifying 21-deoxycortisol (21-DOC), 17-hydroxyprogesterone (17-OHP), cortisol, and cortisone in human plasma has been developed using ultra-high performance liquid chromatography coupled with tandem mass spectrometry (UHPLC-MS/MS). Analysis of plasma samples were performed on Atlantis dC18 (3 *μ*m) column using a mobile phase of 20.0 mM ammonium acetate and acetonitrile (50:50, *v* : *v*) that was delivered at isocratic flow rate 0.3 mL/minute. After addition of d4-cortisol as an internal standard (IS), plasma samples containing 21-DOC, 17-OHP, cortisol, and cortisone were extracted with mixture of dichloromethane and tert-butylmethyl ether 1:2 (*v*/*v*). Analytes were detected and quantified in the positive ion mode of electrospray ionization using multiple reaction monitoring transition set at mass to charge (m/z): 347.17 ⟶ 311.12, 331.17 ⟶ 96.97, 363.11 ⟶ 121.00, 361.18 ⟶ 163.11, and 367.19 ⟶ 121.24 for 21-DOC and 17-OHP, cortisol, cortisone, and cortisol-d4 (IS), respectively. The relationship between concentration and peak area response (analyte/IS) were linear over the range of 0.25–50, 0.5–100, 1–200, and 2–400 ng/mL for 21-DOC, 17-OHP, cortisone, and cortisol, respectively. The mean extraction recovery of the analytes was in the range of 83%–96%. The coefficient of variation within and between days was less than 13.6%, and the bias ranged from −9.2% to 12%. The measured level of cortisol, cortisone, and 17-OHP was in the range of 21.9–110, 4.33–12.71, and 0.37–1.4 ng/mL, respectively. Furthermore, the measured value of cortisone–cortisol ratio was in the range of 0.08–0.21.

## 1. Introduction

Congenital adrenal hyperplasia (CAH) is a genetic disorder caused by 21-hydroxylase deficiency in androgen excess diseases originating in the adrenal gland. Approximately 95% of CAH cases worldwide are brought on by a 21-hydroxylase deficiency. The most prevalent type of CAH is brought on by a pathogenic variation in the gene that codes for the 21-hydroxylase enzyme (CYP21A2) [1]. For many years, the gold standard test for CAH screening and diagnosis was 17-hydroxyprogesterone (17-OHP) [1, 2]. Conversely, 17-OHP is elevated in a number of CAH causes, such as *P*450 oxidoreductase, 3-beta-hydroxysteroid dehydrogenase, and 11-hydroxylase impairment. Furthermore, it is higher in both premature and term infants who are under stress (around 40%) of samples that were first classified as positive will later have normal 17-OHP levels when evaluated by liquid chromatography-mass spectrometry (LC-MS/MS) [3].

Due to 21-hydroxylase deficiency in CAH, the preferred test is 21-deoxycortisol (21-DOC), which is formed as an intermediary steroid in the glucocorticoid pathway [4, 5]. The accuracy of newborn screening for 21-hydroxylase deficiency can be enhanced by the use of plasma 21-DOC, an effective marker of this condition. Numerous analytical techniques have been reported for measuring 21-DOC alone or in combination with other corticoids. These techniques include radio immunoassays (RIAs) [6, 7], enzyme-linked immunosorbent assays (ELISA) [8], micellar electrokinetic capillary chromatography (MEKC) [9], gas chromatography mass spectrometry (GC-MS) [10], high performance liquid chromatography (HPLC) [11], LC-MS/MS [12–14], and LC-MS/MS combined with differential ion mobility spectrometry [15].

The use of immunoassay techniques to detect 21-DOC may result in false-positive readings due to substance cross-reactions. Thus, Zhang et al. described a method that involves steroid purification by HPLC prior to RIA analysis in order to avoid interferences [16], whereas micellar electrokinetic capillary electrophoreses has been shown highly effective technique for quantifying this hormone. However, the sensitivity was deemed insufficient in the absence of preconcentration [17]. A number of LC-MS/MS methods that have been reported for the measurement of four or more steroids simultaneously are based on utilization solid-phase extraction procedures in sample preparation [12–14], which raises the cost and complexity of analysis. Recently, Terabe and Kim described a UHPLC-MS/MS method that concurrently detects 17 corticosteroid hormones in human plasma using 15 different isotopic internal standards [18]. However, it is frequently noted that in a typical commercial situation, it is challenging to obtain compounds with an isotopic label.

In this study, we present an UHPLC-MS/MS assay that offers four distinct advantages, including easy use of internal standards, simultaneous measurement of essential corticosteroids, rapid and efficient analysis, cost-effectiveness, and accessibility.

## 2. Methodology

### 2.1. Chemicals and Reagents

The 21-DOC, 17-OHP, cortisol, cortisone, and cortisol-d4 (IS) purchased from Sigma-Aldrich MO. USA. Ammonium acetate, methanol, and acetonitrile (HPLC grade) were purchased from Fisher Scientific, NJ. USA. Ultrapure water (Milli-Q) was prepared by passing purified water through the system (Millipore, Bedford, MA, USA). The study was conducted after the approval of Research Ethics Committee, King Faisal Specialist Hospital & Research Center, Riyadh, Saudi Arabia, under Research Advisory Council (RAC# 2230001).

### 2.2. Instrument and Chromatographic Conditions

The UHPLC-MS/MS consisted of Xevo-TQD detector equipped with *Z*-spray, an atmospheric pressure ionization (API) interface, Acquity UPLC H-Class system, and integrated solvent and sample manager (Waters Corporation, Milford, MA, USA). Analysis was performed at room temperature using a reversed phase Atlantis dC18 column (2.1 × 100 mm, 3 μm) steel column, protected by an on-line guard column filter (0.2 μm × 2 mm). The mobile phase was composed of 20 mM ammonium acetate and acetonitrile (50:50, *v*/*v*) and was delivered at an isocratic flow rate of 0.3 mL/min with run time 5 minutes. The electrospray ionization (ESI) source operated in the positive-ion mode at a capillary voltage of 1.50 kV and a cone voltage of 35 V. Nitrogen was used as the nebulizing and desolvation gas at a flow rate 1000 L/hr. Argon was used as the collision gas at a pressure of 3.6 × 10^−3^ mbar. The optimum collision energy for 21-DOC, 17-OHP, cortisol, cortisone, and the IS was 20 eV. The ion source and the desolvation temperatures were maintained at 150°C and 500°C, respectively. The 21-DOC, 17-OHP, cortisol, cortisone, and IS were detected and quantified in the positive ion mode; product ion response was measured at mass to charge (m/z): 347.17 ⟶ 311.12, 331.17 ⟶ 96.97, 363.11 ⟶ 121.00, 361.18 ⟶ 163.11, and 367.19 ⟶ 121.24, respectively. Mass lynx Ver 4.1 (Waters Corporation, Milford, MA, USA) software working under Microsoft Window XP professional environment was used to control the instrument parameters, data acquisition, peak integration, peak smoothing, and signal-to-noise ratio measurements.

### 2.3. Standard and Control Sample Preparation

The 21-DOC, 17-OHP, cortisol, cortisone, and IS stock solutions were prepared in methanol (1.0 μg/mL). Utilizing stock solution, calibration curve and control samples were prepared in human plasma. Nine points' calibration curve samples were prepared for 21-DOC, 17-OHP, cortisol, and cortisone, respectively, in the ranges of 0.25–50, 0.5–100, 2.0–400 ng/mL, and 1.0–200 ng/mL. Three control samples at level (low [3 × lower limit of quantification, LOQ], medium [0.5 × upper limit of quantification, ULQ] and high [0.9 × ULQ]) were prepared. All the solutions stored at −20°C until used within eight weeks.

### 2.4. Sample Collection

Twenty-two human plasma samples (2-3 mL each) collected from healthy adults were analyzed to assess the method's efficacy. The study was conducted in accordance with the Declaration of Helsinki and approved by the King Faisal Specialist Hospital & Research Center's Research Ethics Committee in Riyadh, Saudi Arabia.

### 2.5. Processing of Plasma Samples

A total of 300 μL aliquots plasma of calibration standard, quality control (QC), or test samples were placed in 1.5 mL microcentrifuge tube. To each tube, 50 μL of the deuterated IS working solution (100 ng/mL in methanol) was added and then vortexed for 20 s. After the addition of 900 μL of solvent mixture (dichloromethane and tert-butylmethyl ether, 1:2, *v*/*v*), samples were vortexed again for 30 s and then centrifuged 10 min at 1100 rpm at room temperature. The organic layer was carefully collected into a clean borosilicate glass tube and evaporated to dryness under a gentle steam of nitrogen on multiplace heating block at temperature not exceeding 40°C; the residue was reconstituted in 75 μL methanol and then centrifuged at 8400 rpm for 5 min at room temperature. The supernatant was transferred into an autosampler vial, and 10 μL clear solution was injected into the UHPLC-MS/MS system.

### 2.6. Extraction Recovery

An extraction method has been developed for the measurement of 21-DOC, 17-OHP, cortisol, and cortisone in human plasma. It involves liquid–liquid extraction, followed by UHPLC with tandem mass spectrometry detection. Ethyl acetate, dichloromethane, and tert-butylmethyl ether were the three solvents we first employed to investigate their suitability for isolating these components from human plasma. Comparing recovery results, however, reveals that the best extraction solvent that provided consistent and reliable outcomes was a mixture of dichloromethane and tert-butylmethyl ether (1:2, *v*/*v*). The mean of five measurements was used to determine each extraction recovery by comparing the peak area response of the analytes at three concentrations pre- and post-spike blank plasma extract. The extraction recovery of each was calculated as mean of five measurements by comparing peak area response of analytes pre-spike blank plasma and postspike blank plasma extract at three concentrations. The concentrations are (0.75, 25, and 45 ng/mL), (1.5, 50, and 90 ng/mL), (6.0, 200, and 360 ng/mL), and (3, 100, and 180 ng/mL) for 21-DOC, 17-OHP, cortisol, and cortisone, respectively. Similarly, the recovery of deuterated IS was determined at single concentration (100 ng/mL).

### 2.7. Effect of the Matrix

Matrix effect was evaluated by comparing peak area response obtained from post-spike blank plasma extract and standard samples prepared in methanol at three concentrations of (0.75, 25, 45 ng/mL), (1.5, 50, 90 ng/mL), (6.0, 200, 360 ng/mL), and (3, 100, 180 ng/mL) for 21-DOC, 17-OHP, cortisol, and cortisone, respectively. In contrast, the matrix effect on deuterated IS was assessed at a single concentration prepared with 100 ng/mL of methanol.

### 2.8. Stability Studies

For stability investigations, two QC samples of each analyte at low and high plasma concentrations were utilized. Each sample was extracted into five aliquots, of which five were immediately tested (baseline), five were held at −20°C for eight weeks, and five were let to stand at room temperature on the bench for an entire day before being processed and analyzed. Furthermore, prior to analysis, five aliquots were prepared and kept at room temperature for 24 h or at −20°C for 48 h. Lastly, each sample was kept for 24 h at −20°C in 15 aliquots. Five aliquots were examined after being allowed to fully thaw naturally at ambient temperature, while the other aliquots were kept at −20°C for another day.

## 3. Results and Discussion

### 3.1. Method Development

**Figure 1:**
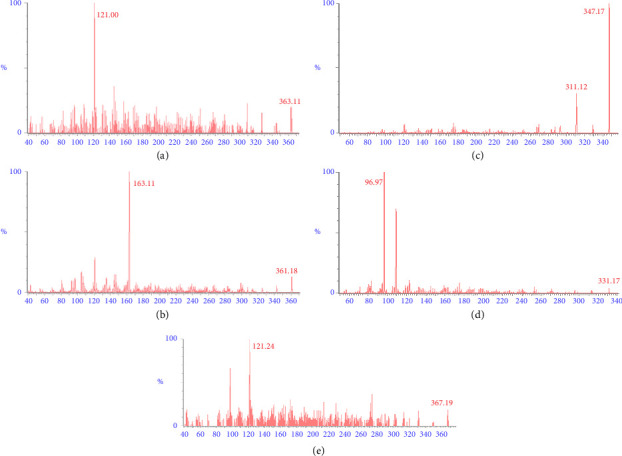
MS/MS spectra of the analytes under studies: (a) cortisol, (b) cortisone, (c) 21-deoxycortisol, (d) 17-hydroxyprogesterone, and (e) cortisol-d4 (IS).

**Figure 2:**
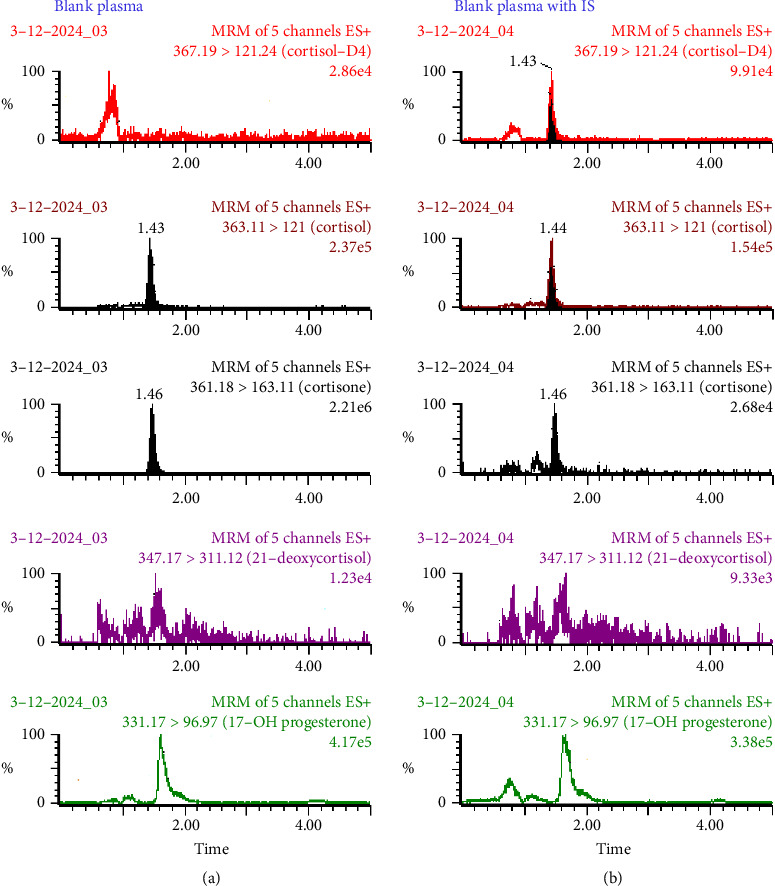
MRM chromatogram of blank plasma (a) and blank plasma spike with 5.0 ng cortisol-d4 as internal standard (b).

The standard mixture solution containing 21-DOC, 17-OHP, cortisol, cortisone, and the IS (1.0 *μ*g/mL each) was injected using an infusion pump at a flow rate of 20 *μ*L/mL in order to optimize the parameters of the MS/MS method. The mass to charge (m/z) transitions for precursor and product ions were set at 347.17 ⟶ 311.12, 331.17 ⟶ 96.97, 363.11 ⟶ 121.00, 361.18 ⟶ 163.11, and 367.19 ⟶ 121.24 for 21-DOC and 17-OHP, cortisol, cortisone, and IS, respectively, as illustrated in Figure 1. UHPLC-MS/MS method-optimized parameters are given in Table 1. The developed method's parameters are on the line of reported assay for cortisol measurement in saliva [19]. The mobile phase consisted of 20 mM ammonium acetate and acetonitrile (50:50, *v* : *v*) operated under isocratic conditions at room temperature in order to optimize UHPLC settings. The multiple reaction monitoring (MRM) chromatogram of plasma extract for each analyte at the upper limit of quantitation: (a) cortisol (400 ng/mL), (b) cortisone (200 ng/mL), (c) 21-DOC (100 ng/mL), and (d) 17-OHP (50 ng/mL) spiked with internal standard (50 μL) of 100 ng/mL solution are shown in Figure 2. An isotopically labeled internal standard is usually used for each analyte in a quantitative experiment to control variability. However, we used one isotopically labeled internal standard to assess all four analytes, which produced the highest bias (±12.7%) and precision (11.3%), respectively. Both levels remained within the acceptable ranges specified bioanalytical method validation guidelines by USFDA [20].

The newly developed method's main advantages are affordability, ease of use of internal standards, rapid and efficient analysis, and simultaneous assessment of four clinically significant corticosteroids: cortisol, cortisone, 17-OHP, and 21-DOC.

### 3.2. Matrix Effect and Recovery

In order to assess the matrix effect, we used peak areas of post-spiked blank plasma and compared with peak areas of standards prepared in methanol as per the method. No significant matrix effect was observed. However, the measured effect was as calculated as ion suppression (≤ 8.6%). Extraction recovery of 21-DOC, 17-OHP, cortisol, cortisone, and the IS from plasma was assessed using five replicates of three concentrations of each analyte at each level (low, medium, and high) and one concentration of the IS (100 ng/mL). Measured mean extraction recovery was in the range of (84%–97%) 21-DOC, (85%–88%) 17-OHP, (84%–98%) cortisone, (85%–98%) cortisol, and (93%) IS, respectively.  Table 2 depicts the results that were obtained, indicating that all of the measurements remain within the permissible range according to the USFDA's requirements (matrix effect < 15% and recovery must be consistent).

### 3.3. Method Validation

To ensure the method's validity, the parameters such as specificity, range, extraction recovery, matrix effect, precision, and accuracy were examined according to international reference standards, [20].

### 3.4. Specificity

**Figure 3:**
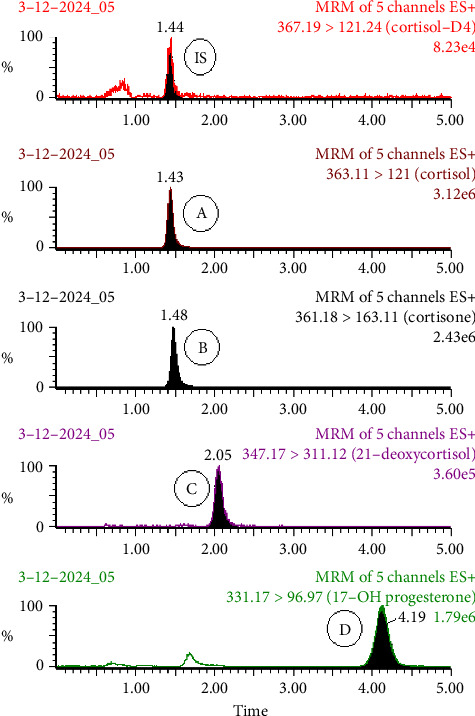
MRM chromatogram of plasma extract of each analyte at the upper limit of quantitation: (A) cortisol (400 ng/mL), (B) cortisone (200 ng/mL), (C) 21-deoxycortisol (100 ng/mL), and (D) 17-hydroxyprogesterone (50 ng/mL) spiked with (IS) internal standard (50 μL) of 100 ng/mL solution.

The method's specificity was tested by evaluating six different blank human plasma samples and six structurally related steroids that may be found in blank biological fluids, including testosterone, prednisolone, methylprednisolone, progesterone, and prednisone. All solutions were prepared in methanol: water (1:1, *v* : *v*) at a concentration of 1.0 μg/mL, and 10 μL was introduced into the system. The peaks of the analytes were found to be free of interference. In addition, to assess the interference in the simultaneous measurement of four analytes, we prepare and analyze each analyte individually at upper limit of quantification to verify the presence of other analytes. It had been verified that there was no interference.  Figure 3 depicts the chromatogram samples analyzed. However, we were unable to examine its impact on analyte measurements due to the unavailability of hemolyzed and lipemic plasma.

### 3.5. Linearity, Limit of Detection, and Quantification

Standard mixtures containing 21-DOC, 17-OHP, cortisone, and cortisol in human plasma at nine different concentrations in range of (0.25–50 ng/mL), (0.5–100 ng/mL), (1.0–200 ng/mL), and (2.0–400 ng/mL) were evaluated to determine the assay's linearity. A linear regression equation (*y* = *mX* + *c*), where *X* is an independent variable and *Y* is dependent variable. The slope of the curve (*m*) and (*c*) is the intercept used to examine the peak area ratios and the corresponding concentrations. The coefficient of determination (*r*^2^) was ≥ 0.9894. The limit of detection (0.15, 0.30, 0.25, and 0.30) ng/mL and quantification (0.25, 0.50, 1.0, and 2.0) ng/mL for 21-DOC, 17-OHP, cortisone, and cortisol were established based on signal-to-noise ratio response three and five, respectively. The linearity data that include measurement ranges and inaccuracy (bias) and precision (CV) calculated based on six calibration curves' data are shown in Table 3.

### 3.6. Precision and Accuracy

Three quality control samples containing 21-DOC (0.75, 25, 45 ng/mL), 17-OHP (1.5, 50, 90 ng/mL), cortisone (3,100, 180 ng/mL), and cortisol (6, 200, 360 ng/mL) in human plasma were evaluated to determine the assay's precision and accuracy. Intraday (*n* = 10) precision was measured as coefficient of variation (CV, %) within the range 2.4–11.3, whereas accuracy was measured as bias (%) not more than ±12.7%. Interday (*n* = 20) precision and bias, determined over 3 days, were ≤ 10.5% and the bias ranged from −9.7% to 6.6%, respectively. Data are summarized in Table 4 which indicate that the precision (coefficient of correlation, CV) and inaccuracy (bias) measured values remain within the acceptable range, in accordance with USFDA guidelines for bioanalytical methods.

### 3.7. Stability

The 21-DOC, 17-OHP, cortisol, and cortisone stability in processed and unprocessed plasma samples at two concentrations, low (3xLOQ) and high (0.9xHOQ), were investigated. The 21-DOC, 17-OHP, cortisol, and cortisone were stable in processed samples for at least 24 h at room temperature (≥ 97%) whereas in unprocessed plasma samples, it is stable for at least 24 h at room temperature (≥ 94), at least eight weeks at −20°C (≥ 91%) and after three freeze-and-thaw cycles (≥ 92%).

### 3.8. Assay's Application

**Figure 4:**
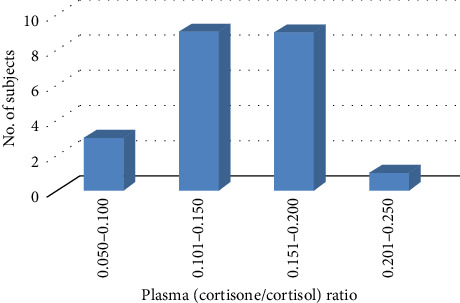
Plasma cortisone–cortisol ratio in samples collected from healthy volunteers.

The method used to determine the level of cortisol, cortisone, 17-OHP, and 21-DOC in plasma samples (22) collected from a healthy volunteers. The measured level was in the range of (21.9–110 ng/mL), (4.33–12.71 ng/mL), and (0.37–1.4 ng/mL) for cortisol, cortisone, and 17-OHP, respectively.  Figure 4 represents the interconversion of cortisol–cortisone through isoenzyme 11-beta hydroxysteroid hydrogenase-2, which regulates cortisol metabolism. The cortisone–cortisol ratio was found to be measured within the range of 0.08–0.21, which is consistent with the usual range of 0.081–0.301 [21]. In routine practice, newborn screening the measurement of 21-DOC can supplement or confirm 17-OHP in the diagnosis of difficult cases of CAH. When assessing challenging cases of CAH thought to be caused by CYP21A2 impairment, measures of 21-DOC can be used to supplement or validate results of 17-OHP and androstenedione.

When compression to recent study reported by Held et al. [4] to evaluate the influence of gestational age and the timing of collection on 21-DOC concentrations in 906 new born screen specimens confirmed that the 21-DOC cutoff value 0.85 ng/mL yielded a 91.7% positive prediction value for 21-hydroxylase deficiency. The method we present here can be used to measure 21-DOC levels that are below the cutoff value in the range of 0.25–50 ng/mL.

## 4. Conclusion

The validated assay successfully used for simultaneous measurement of 21-DOC, 17-OHP, cortisol, and cortisone in of plasma samples obtained from healthy controls. Assay was utilized to evaluate cortisone/cortisol ratio of individual that provides a clinical clue in assessing the activity of isoenzyme (11ß-hydroxysteroid hydrogenase-2). Assay may be used as secondary test for confirmation of congenital adrenal hyperplasia in patient samples.

### 4.1. Assay Limitations

A potential limitation of this study is the absence of CAH samples. While the developed method has shown excellent performance in measuring key corticosteroids in plasma, its application to CAH patients, a condition characterized by abnormal corticosteroid levels, was not evaluated in this study. The inclusion of CAH samples would be valuable for further validating the method's clinical applicability in diagnosing and monitoring adrenal disorders. This remains an important direction for future research.

## Figures and Tables

**Table 1:** LC-MS/MS parameters and peak identification.

Analyte	Retention time (min)	Molecular mass (M)	Parent ion (M + H)	Fragment ion (Major)	Cone voltage (kV)	Capillary energy (V)
Cortisol	1.43	362.46	363.11	121.00	30	22
Cortisone	1.48	360.45	361.18	163.11	38	24
21-deoxycortisol	2.05	346.46	347.17	311.12	36	14
17-hydroxyprogesterone	4.19	330.46	331.17	96.97	36	26
Cortisol-d4 (IS)	1.44	366.46	367.19	121.24	34	24

**Table 2:** Extraction recovery and matrix effect.

Analyte/Concentration (ng/mL)	⁣^∗∗^Mean peak area (SD)			⁣^∗^Recovery (%)	^†^Matrix Effect (%)
	Analyte spiked plasma extract (A)	Plasma extract analyte spiked (B)	Standards methanol (C)		
21-Deoxycortisol					
0.75	702 (55)	834 (62)	977 (69)	84	−8.2
25	7846 (146)	8239 (345)	8712 (196)	95	−5.4
45	14,311 (249)	14,799 (560)	15,613 (497)	97	−5.2
17-Hydroxyprogesterone					
1.5	9400 (584)	11,002 (585)	11,193 (416)	85	−1.7
50	64,602 (1518)	74,640 (1362)	78,422 (1406)	87	−5.1
90	115,049 (4668)	130,924 (4929)	141,074 (7778)	88	−7.2
Cortisone					
3.0	15,441 (389)	15,809 (423)	16,005 (517)	98	−1.2
100	54,478 (994)	63,336 (1963)	69,269 (5598)	86	−8.6
180	126,564 (2936)	150,805 (6034)	159,967 (5057)	84	−5.7
Cortisol					
6.0	14,610 (584)	16,178 (388)	16,508 (819)	90	−2.0
200	86,593 (2919)	88,168 (2958)	93,672 (4845)	98	−5.9
360	154,470 (9719)	182,725 (2667)	198,128 (3738)	85	−7.8

Abbreviation: SD, standard deviation.

⁣^∗∗^Mean (*n* = 5).

⁣^∗^Recovery (%) = A/B × 100.

^†^Matrix effect (%) = (B−C)/C × 100.

**Table 3:** Linearity data.

Analyte	Concentration range (ng/mL)		Coefficient correlation (*r*^2^)	Bias (%) (min–max)	CV (%) (min–max)	Mean regression equation (*n* = 6)
	Min	Max				
21-DOC	0.25	50	0.9963	−4.4–14.0	1.8–15.5	*y* = 0.0592*x* + 0.0009
17-OHP	0.50	100	0.9949	−8.3–13.0	2.8–10.9	*y* = 0.2468*x* − 0.0531
Cortisone	1.00	200	0.9942	−5.1–4.8	2.7–11.0	*y* = 0.1168*x* + 0.7351
Cortisol	2.00	400	0.9943	−7.6–7.3	2.2–10.5	*y* = 0.0694*x* + 0.0508

Abbreviations: LOD = limit of detection, LOQ = limit of quantification, UOQ = upper limit of quantification.

⁣^∗^Replicate analysis (*n* = 6).

**Table 4:** Intra- and interday precision and accuracy of the assay.

Nominal level (ng/mL)	Intraday (*n* = 10) measured level		Interday (*n* = 20) measured level			
	Mean (SD)	CV (%)	Bias (%)	Mean (SD)	CV (%)	Bias (%)
Cortisol						
6.0	5.99 (0.44)	7.3	0.2	6.01 (0.46)	7.7	6.9
200	191.23 (21.59)	11.3	−4.4	196.8 (20.63)	−1.6	−1.6
360	319.42 (12.55)	3.9	−11.3	336.14 (25.83)	6.6	6.6
Cortisone						
3.0	2.89 (0.19)	6.7	3.5	2.92 (0.22)	7.5	−2.6
100	102.01 (6.23)	6.1	2.0	101.67 (9.33)	9.2	1.7
180	185.04 (8.85)	4.8	2.8	180.63 (9.84)	5.5	0.4
17-OHP						
1.5	1.54 (0.15)	9.5	2.4	1.45 (0.15)	10.5	−3.3
50	47.70 (4.90)	10.4	−4.6	47.4 (4.40)	9.2	−5.2
90	80.12 (2.63)	3.3	−11.0	82.80 (5.28)	6.4	−8.0
21-DOC						
0.75	0.78 (0.06)	7.3	3.7	0.76 (0.06)	7.2	1.8
25	23.11 (1.94)	8.4	−7.5	22.98 (1.59)	6.9	−8.1
45	39.27 (0.93)	2.4	−12.7	40.65 (2.27)	5.6	−9.7

*Note:* CV, coefficient of variation = standard deviation divided by mean measured concentration × 100. Bias = measured level−nominal level divided by nominal level × 100.

Abbreviation: SD, standard deviation.

## Data Availability

The data that support the findings of this study are available from the corresponding author upon reasonable request.
